# Early Developmental Low-Dose Methylmercury Exposure Alters Learning and Memory in Periadolescent but Not Young Adult Rats

**DOI:** 10.1155/2016/6532108

**Published:** 2016-01-13

**Authors:** Damaris Albores-Garcia, Leonor C. Acosta-Saavedra, Alberto J. Hernandez, Miriam J. Loera, Emma S. Calderón-Aranda

**Affiliations:** Departamento de Toxicología, CINVESTAV, 07360 México, DF, Mexico

## Abstract

Few studies have assessed the effects of developmental methylmercury (MeHg) exposure on learning and memory at different ages. The possibility of the amelioration or worsening of the effects has not been sufficiently investigated. This study aimed to assess whether low-dose MeHg exposure* in utero* and during suckling induces differential disturbances in learning and memory of periadolescent and young adult rats. Four experimental groups of pregnant Sprague-Dawley rats were orally exposed to MeHg or vehicle from gestational day 5 to weaning: (1) control (vehicle), (2) 250 *μ*g/kg/day MeHg, (3) 500 *μ*g/kg/day MeHg, and (4) vehicle, and treated on the test day with MK-801 (0.15 mg/kg i.p.), an antagonist of the N-methyl D-aspartate receptor. The effects were evaluated in male offspring through the open field test, object recognition test, Morris water maze, and conditioned taste aversion. For each test and stage assessed, different groups of animals were used. MeHg exposure, in a dose-dependent manner, disrupted exploratory behaviour, recognition memory, spatial learning, and acquisition of aversive memories in periadolescent rats, but alterations were not observed in littermates tested in young adulthood. These results suggest that developmental low-dose exposure to MeHg induces age-dependent detrimental effects. The relevance of decreasing exposure to MeHg in humans remains to be determined.

## 1. Introduction

Developmental exposure to environmental pollutants has been associated with the onset of cognitive disturbances, due to the sensitivity of the immature central nervous system (CNS) to external insults. Methylmercury (MeHg) is a global pollutant with known effects on the CNS, especially when the exposure occurs at early developmental stages, as demonstrated in the Minamata and Iraq episodes [1, 2]. Recently, epidemiological studies evaluated the neurocognitive outcomes of MeHg exposure on fish-eating populations [3–5] and non-fish-eating populations [6], without conclusive results. In America, MeHg contamination is a public health problem [7–12]; Trasande et al.'s study [13] suggested that the annual impact of MeHg exposure in the U.S. reaches nearly half a million children born with cord blood mercury levels associated with a diminishment of Intelligence Quotient (IQ), which could be detrimental to economic productivity. The possible monetary benefit of reducing MeHg exposure in the European population, related to IQ outcomes, has also been reported [14]. Data from experimental studies suggests that neonatal exposure to low-doses of MeHg is associated with visual, memory, and social alterations in nonhuman primates [15–17], memory deficits, and depressive-like behaviour in mice [18] and at higher doses (>3,000 *μ*g/kg/day) severe motor dysfunction and cognitive deficits [19]. Rats, due to their toxicokinetics, must consume 10-fold higher doses of MeHg than humans, nonhuman primates, and mice to achieve similar brain Hg levels and present neurotoxic effects [20, 21]. Thus, to perform an accurate interspecies comparison, it is necessary to consider the dose, exposure schedule, and toxicokinetics of the experimental model. Experimental evidence from pregnant rats exposed to daily doses of 500 *μ*g/kg of MeHg showed that MeHg levels in the brain of pups reached concentrations in the range of MeHg levels found in the brains of infants from populations exposed through fish consumption [20, 22].

Severe adverse effects on learning and memory have been reported in experiments using large doses of MeHg (1,000 to 8,000 *μ*g/kg/day) and different exposure schedules in rodents [23–25]. Studies that have used doses of MeHg under 1,000 *μ*g/kg/day have found neurocognitive effects ranging from nonexistent to subtle disturbances [18, 26–29], but few studies have evaluated the effects of MeHg exposure during gestation and lactation on learning and memory at different ages. Sakamoto et al. [26] found alterations in motor coordination and learning disabilities on the passive avoidance test at 5 and 6 weeks of age in rats exposed throughout gestation and until 2 months old. Kakita et al. [30] found a decrease in the neuron population of the amygdala and hippocampus but not the dentate gyrus and learning disabilities in the passive avoidance test and unaffected spatial learning in 6-month-old rats exposed to 1,000 *μ*g/kg/day of MeHg during gestation. Onishchenko et al. [18] found a decrease in reference memory and depressive-like behaviour in transgenic ARE-hPAP mice gestationally exposed to 500 *μ*g/kg/day of MeHg at 5–15 and 26–36 weeks old. Weiss et al. [31] found adverse effects on motor coordination in mice that were exposed throughout their lives to 1,000 or 3,000 *μ*g/kg/day of MeHg. Therefore, it is important to determine whether gestational exposure to low-doses of MeHg produce age-dependent effects on learning and memory, to support studies in humans and evaluate actions to reduce exposure to MeHg and to apply early interventions to decrease the long-term harmful effects of MeHg.

Different types of learning and memory tasks for rodents have been used to evaluate the effects of toxicants and the participation of specific brain structures in these processes. The open field test (OFT) [32] has been used to evaluate the effect of MeHg on locomotor activity. The object recognition test (ORT), the Morris water maze (MWM), and conditioned taste aversion (CTA) [32–34] have been used to evaluate the potentially deleterious effects of MeHg on learning and memory because these experimental tools provide information about different aspects of the processes of interest. Thus, we used these tools with the aim of examining whether exposure to low-doses of MeHg (250 and 500 *μ*g/kg/day) in rats during gestation and lactation was capable of inducing disturbances in learning and memory at different life stages; littermates were tested on postnatal day- (PND-) 40 (periadolescence) and on PND-90 (young adulthood). In animal models, doses that lead to Hg brain concentrations up to 3 *μ*g/g have been referred to as low Hg doses [35]. The higher dose used in our study (500 *μ*g/kg/day) leads to brain concentrations of Hg that are below this level, according to previous reported data [26, 36]. We used a rat model exposed to low-doses of MeHg from gestational day (GD) 5 to PND-21 (weaning); the offspring were exposed through the mother, by placental and milk transfer. The exposure period, doses, and administration routes were chosen to resemble the occurrence of exposures in humans during development; in addition, we used a drug-treated group (MK-801) as a positive control for detrimental effects on learning and memory; this drug is a noncompetitive antagonist of the N-methyl D-aspartate receptor and was used because of its known effects as a performance inhibitor for all tasks tested in this study.

## 2. Methods

### 2.1. Animals

We used Sprague-Dawley primiparous female rats weighing 220–250 g (Harlan, Mexico), which remained in the CINVESTAV animal care facility. The rats were maintained under a light-dark cycle of 12 h/day, in a room with controlled temperature (22°C), humidity (45–50%), and free access to food and water. Two female rats were placed with single male rats, and vaginal smears were taken the next morning. Sixty pregnant rats were randomly assigned to eight groups and these were submitted to four experimental conditions, using two groups per experimental condition.

### 2.2. Chemicals

Methylmercuric chloride (methylmercury; 95% purity) was obtained from Sigma (Sigma-Aldrich, St Louis, MO, USA). A solution of 750–1,500 *μ*g/mL of MeHg was prepared daily in sterile deionized water. MK-801 was obtained from Sigma (Sigma-Aldrich, St Louis, MO, USA) and dissolved in sterile water.

### 2.3. Dosing

Pregnant rats were treated from GD-5 until weaning at PND-21; rats were weighed daily before the administration of the treatments. For control and MK-801 groups 150 *μ*L of water was orally administered; for groups exposed to MeHg the final volume per rat was in a range of 100–150 *μ*L, which was orally administered to achieve a final dose of 250 or 500 *μ*g/kg/day. MK-801 was administered prior to behavioural tasks (0.15 mg/kg, i.p. 15 min before the trial). Control and MeHg-treated groups received saline solution (i.p.) 15 min before the trial. In dams, no evidence of toxicity, motor alterations, abortions, or general health disturbances was observed during gestation and lactation; additionally, the litter size of MeHg dams was not different from the control group. No differences in body weight between pups of different experimental groups were observed.

### 2.4. Behavioural Testing

**Figure 1:**
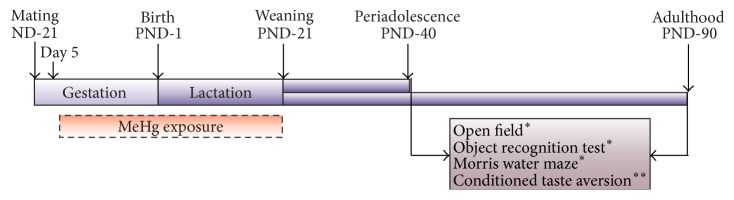
Experimental design. Pregnant rats were orally exposed to MeHg (250 or 500 *μ*g/kg/day) from gestational day (GD) 5 to weaning (postnatal day- (PND-) 21). All exposure to MeHg was stopped after weaning. The learning and memory tasks were performed at PND-40 (periadolescence) and PND-90 (young adulthood), using a different cohort of rats for each task and evaluation time. ^*∗*^ *n* = 10 per experimental group; ^*∗∗*^ *n* = 8 per experimental group.

Male pups from each group were randomly assigned to experimental subgroups. Each experimental group included a single male from each litter. The learning and memory tasks were performed at PND-40 and PND-90, with 8 to 10 rats for each experimental condition (control, MK-801, MeHg 250 *μ*g/kg/day, MeHg 500 *μ*g/kg/day) (Table 1 and Figure 1). Test days for learning and memory evaluation were chosen to determine the effects of the low-dose MeHg exposure schedule in periadolescent (PND-40) and young adult rats (PND-90). For each test and stage assessed, different groups of animals were used. In the test room, controlled conditions were maintained for sound, light, humidity, and temperature (22°C). All tests were performed in the light phase of the day (between 9 am and 3 pm). The order in which the animals were trained was randomized. The protocol used in this study was revised and approved by the CINVESTAV Animal Care and Use Committee, avoiding animal suffering at every stage of the experiment.

### 2.5. Open Field Test

The test was conducted as described in the literature [32] with minor modifications. The open field consisted of a square arena (60 cm × 60 cm), surrounded by 40 cm high white walls. Ten rats from each experimental condition were evaluated at PND-40, and a different cohort was evaluated at PND-90. The test began by placing a single rat in the middle of the arena, and its activity was recorded for 5 min. Test session recordings were analysed offline using Any-Maze software (Stoelting, USA). The field was carefully cleaned with 70% ethanol between each rat. The parameters analysed to evaluate locomotor activity in the open field were total distance travelled and number of rearings.

### 2.6. Object Recognition Test

The test was performed as described in the literature [37] with minor changes. Ten rats from each group were evaluated at PND-40, and a different cohort was evaluated at PND-90. Each rat was allowed to explore the empty cage for 5 min for five consecutive days before the test. On the test day, each rat was placed in a cage (60 × 60 cm) with a black floor. The objects to be discriminated were cubes, pyramids, and cylinders, all made from the same material and immovable when located in the field. The test consisted of two trials with a rest period of 90 min between trials. In the first trial (5 min), there were two identical objects in opposite corners of the cage. In the second trial, one new object replaced one of the objects that had been previously shown, and the rat was allowed to explore the object for 5 min. The order of presentation of the objects and their corner location were counterbalanced to avoid bias. These two trials were recorded, and the exploration time for each object was subsequently analysed with Any-Maze software (Stoelting, USA). Exploration was defined as directing the nose toward the object from a distance of 2 cm or closer or touching the object. The recognition index (RI) was used as a measure of the ability of the animal to distinguish new objects from familiar objects and reflected the time that the animal explored the new object compared with the total exploration time.

### 2.7. Morris Water Maze

To analyse spatial memory using the MWM, 10 rats from each experimental condition were evaluated at PND-40, and different groups were used for assays on PDN 90. The task was performed as described in the literature [33] with minor modifications (5 training sessions, 10 assays per day) in a circular tank (180 cm diameter × 70 cm height) filled with water (22°C) to a depth of 40 cm. The pool was divided into four quadrants, and the platform was located in the middle of the west quadrant, 2 cm below the water level. Each animal was given 60 sec to find the hidden platform, and if it failed, it was gently guided to the platform and was allowed to remain there for 30 sec. After that, rats were towel-dried and kept in holding cages for 30 sec between trials. Different distant cues around the room were kept in the same location during the experiments. The retention test, without the hidden platform, was performed one week after the last training session, releasing the animal from a random start position. All trials were recorded on video for further analysis (Any-Maze, Stoelting, USA) to obtain the swim path, average speed, total distance travelled, and the number of crossings over the platform area. Training curve data were normalized using the first day latency as 100% for each experimental group.

### 2.8. Conditioned Taste Aversion

CTA was performed as described in the literature [34] with minor modifications. A group of rats (*n* = 8) from each experimental condition was used for CTA assessment at PND-40, and different groups were used for assays at PND-90. The protocol started with water deprivation 24 h prior to the training. After that, the animals received tap water in their home cages every 24 h for 15 min, and the amount that the animals consumed was measured. When animals reached a stable water consumption (referred to as baseline consumption), they received an acquisition trial; on this day, a new flavour was presented (saccharin 0.1%, Sigma, Mexico), and 15 min after they drank the novel flavour, a malaise-inducing drug was administered i.p. (lithium chloride, LiCl, 0.15 M, 7.5 mL/kg, Baker, Mexico). For the following two days, baseline intake of tap water was reestablished, and for the next five days, animals received saccharin (15 min) followed by water (15 min); these experimental days are represented as Test 1 to Test 5. Data from these experiments are represented by the aversion index (AI), which is a ratio that reflects the amount of saccharin ingested relative to the total liquid intake of the day (1.0 = saccharin + water).

### 2.9. Statistical Analysis

Data reported in the text and figures indicate the mean ± SEM. For the OFT and ORT analyses, one-way ANOVA followed by Bonferroni* post hoc* tests was performed. For MWM and CTA we used two-way ANOVA, using the factors treatment and time (days), followed by Bonferroni* post hoc* tests. Differences were considered statistically significant if the *p* value was less than 0.05. Data analyses were performed using the GraphPad Prism 5.0 software (GraphPad Software, Inc., San Diego, CA) and all analyses were performed offline by an observer who was blinded to the treatments.

## 3. Results

### 3.1. Methylmercury Exposure Subtly Alters Spontaneous Activity

**Figure 2:**
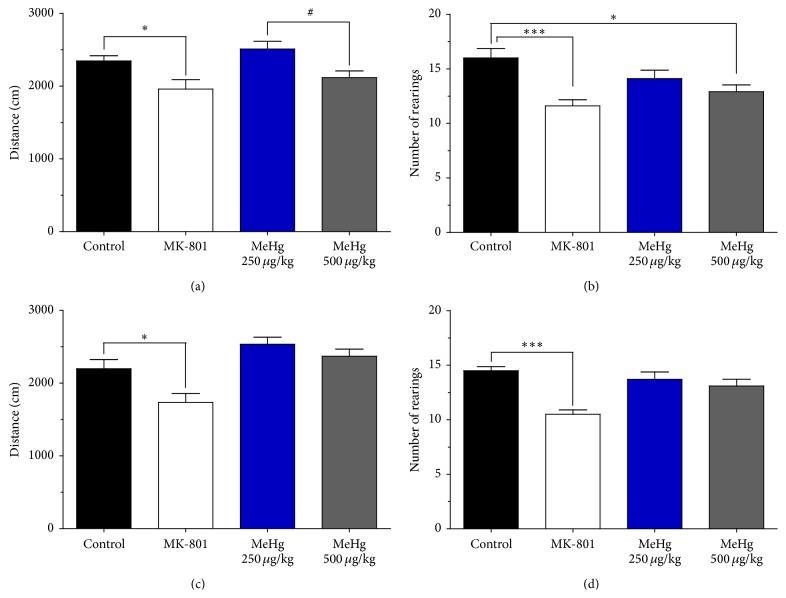
The effects of methylmercury on locomotor and exploratory behaviour. OFT was performed at PND-40 (a-b) and PND-90 (c-d). Graphs (a) and (c) show the total distance travelled (cm), and graphs (b) and (d) show the number of rearings (mean ± SEM) at PND-40 and PND-90, respectively. The data were analysed using one-way ANOVA followed by Bonferroni* post hoc* tests. Significant differences compared with the control group (^*∗*^ *p* < 0.05; ^*∗∗∗*^ *p* < 0.001) or with the group exposed to 250 *μ*g/kg/day MeHg (^#^ *p* < 0.05) are indicated.

To evaluate changes in locomotor behaviour, we used the OFT. The data obtained showed no changes in locomotor activity (evaluated as total distance travelled) in both groups of MeHg-exposed rats compared to the control group, assessed at PND-40 and at PND-90 (Figures 2(a) and 2(c) and Table 2). However, differences in locomotor activity between MeHg-treated groups were observed at PND-40 (*p* < 0.05, 95% confidence interval (CI): 14.2–774) (Figure 2(a)) but were not observed in animals assessed at PND-90 (Figure 2(c)). The group exposed to 250 *μ*g/kg/day of MeHg did not differ from the control group in vertical exploration (rearings) at the two assessed times (Figures 2(b) and 2(d)). In contrast, the group exposed to 500 *μ*g/kg/day of MeHg demonstrated a diminishment in vertical exploration compared to the control group (*p* < 0.05, 95% CI: 0.6–5.5) at PND-40 (Figure 2(b)), which was not observed in animals evaluated at PND-90 (Figure 2(d)), but there were no differences between PND-40 and PND-90 in the two MeHg-exposed groups (Table 2). The group treated with MK-801 was found to have a diminishment in locomotor activity (*p* < 0.05, 95% CI: 31.8–741.1 for PND-40 and 76.7–850.5 for PND-90), as well as a decrease in the number of rearings at the two evaluated ages (*p* < 0.001, 95% CI: 1.9–7.2 for PND-40 and 2.0–6.0 for PND-90).  Table 3 shows the *F*-statistic and *p* values from one-way ANOVA analyses for OFT at PND-40 and PND-90.

### 3.2. Methylmercury Exposure Impaired Short-Term Object Recognition Memory

To evaluate object recognition memory by ORT, the RI was used as a measure of performance. The RI reflected the object exploration by the animal; RI values above 0.5 indicated that the animal spent more time with the novel object (normal performance of a rodent); a value of 0.5 indicated that the animal spent the same amount of time exploring both objects (novel and familiar), and values below 0.5 indicated that the animal spent more time exploring the familiar object.

**Figure 3:**
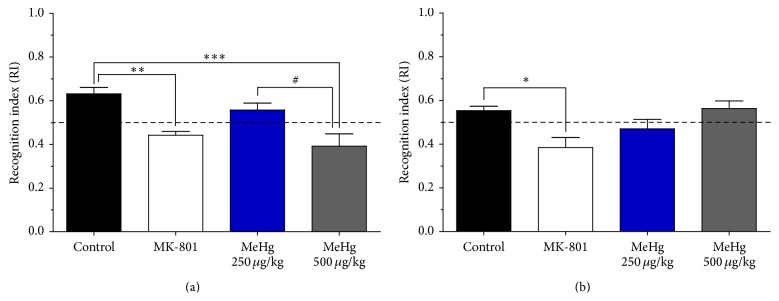
Methylmercury exposure disrupted recognition memory. ORT was performed at PND-40 (a) and PND-90 (b). The graphs show the recognition index (RI: time exploring new object/total exploration time) as the mean ± SEM; an RI below 0.50 indicates that the animal spent more time exploring the familiar object, and an RI above 0.50 indicates that the animal spent more time exploring the new object. The results were analysed using one-way ANOVA and Bonferroni* post hoc* tests. Significant differences compared with the control group (^*∗*^ *p* < 0.05; ^*∗∗*^ *p* < 0.01; ^*∗∗∗*^ *p* < 0.001) or with the group exposed to 250 *μ*g/kg/day MeHg (^#^ *p* < 0.05) are indicated.

The RI indicated that MeHg exposure impaired recognition memory in a dose-dependent manner in animals assessed at PND-40 (Figure 3(a)); this decrement in the RI was statistically significant in the rats exposed to 500 *μ*g/kg/day of MeHg compared to the control group (*p* < 0.001, 95% CI: 0.089–0.21) and compared to the group exposed to 250 *μ*g/kg/day of MeHg (*p* < 0.05, 95% CI: 0.027–0.30). In the groups assessed at PND-90, no differences were observed between the MeHg-exposed animals and control animals or between MeHg-exposed groups (Figure 3(b)). The rats treated with MK-801 showed a statistically significant decrement in the RI compared to the control group at PND-40 (*p* < 0.01, 95% CI: 0.057–0.32) and at PND-90 (*p* < 0.05, 95% CI: −0.33–0.02).  Table 3 shows the *F*-statistics and *p* values from one-way ANOVA analyses for the ORT at PND-40 and PND-90.

The time-course analyses between same-dose treatments did not indicate differences in RI for the 250 *μ*g/kg/day MeHg-treated group at the two developmental windows studied (Table 2). However, the group exposed to 500 *μ*g/kg/day was found to have significant differences in RI between PND-40 and PND-90. Specifically, the RI was higher at PND-90 than at PND-40. This finding indicates that the detrimental effect on recognition memory found at PND-40 (RI was significantly lower than the RI of the control group) was not detected in animals tested at PND-90 (Table 2).

### 3.3. Methylmercury Exposure Altered Spatial Learning

The MWM task reflected the capacity of the animal for spatial learning, exhibited in the rats' undirected swimming in the initial trials and rapid and precise swimming to the hidden platform in the later trials. Training curve data were normalized using the first day latency as 100% for each experimental group because the MeHg-treated rats trained at PND-40 found the hidden platform faster than control animals; however, on the following days, the MeHg-treated animals did not improve their performance. The faster finding of the hidden platform on training day 1 at PND-40 was attributable to faster swimming of MeHg-treated animals (data not shown). The nonnormalized learning curve is shown in supplementary Figure 1 (see Supplementary Material available online at http://dx.doi.org/10.1155/2016/6532108).

**Figure 4:**
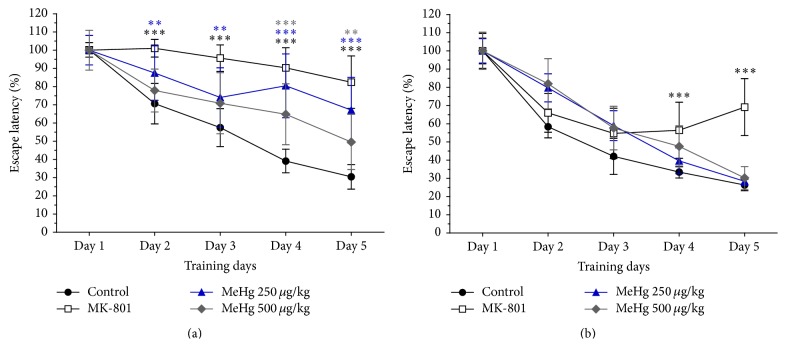
Methylmercury exposure altered spatial learning. Graphs show the learning curve normalized as a percentage of the latency of the group on the first day of training (mean ± SEM) at PND-40 (a) and PND-90 (b). Escape latency results were analysed using two-way ANOVA, followed by Bonferroni* post hoc* tests; statistically significant differences compared with the control group are indicated (^*∗∗*^ *p* < 0.01; ^*∗∗∗*^ *p* < 0.001).

Figure 4 shows the normalized learning curve for control and treated animals day by day, grouping the 10 trials per day for all of the individuals in each group (*n* = 10) trained at PND-40 or at PND-90. For groups assessed at PND-40, the normalized escape latency of control animals started at 100% on day 1 and reached 30% on day 5. Significant differences between the control group and the 250 *μ*g/kg/day MeHg-exposed group were found from days 2 through 5 of training (*p* < 0.01 for days 2 and 3, 95% CI: 1.1–32.5 and 0.8–32.3 for days 2 and 3, resp., and *p* < 0.001 for days 4 and 5, 95% CI: 25.6–57.0 and 20.9–52.3 for days 4 and 5, resp.). The group exposed to 500 *μ*g/kg/day of MeHg was found to have significant differences on training days 4 and 5 compared to the control group (*p* < 0.001, 95% CI: 10.0–41.4 for day 4; *p* < 0.01, 95% CI: 3.4–34.8 for day 5) (Figure 4(a)). The MK-801-treated group exhibited a longer normalized escape latency during training days 2 through 5 (*p* < 0.001, 95% CI: 14.2–46.4, 22.0–54.2, 35.0–67.3, and 35.8–68.0 for days 2, 3, 4, and 5, resp.), which reflected the inability of the animals to learn the task when the NMDAR antagonist was administered at this age (Figure 4(a)). The analysis of within-group differences over time reflects that at PND-40, only control rats learned the task. MK-801-treated animals and both MeHg-exposed groups showed no significant differences in normalized escape latency across the five training days, reflecting the inability of these animals to learn the task (supplementary Figure 2).

In groups trained at PND-90, the rats exposed to 250 and 500 *μ*g/kg/day of MeHg showed no significant differences in normalized escape latency compared to the control group. The MK-801-treated group exhibited a significantly slower learning process than the control group (*p* < 0.001, 95% CI: 7.9–37.9 and 27.8–57.8 for days 4 and 5, resp.) (Figure 4(b)). The analysis of differences over time within groups indicates that, in animals trained at PND-90, control rats learned the task by the second day of training (*p* < 0.01, 95% CI: 12–71); rats exposed to 250 *μ*g/kg/day of MeHg learned the task by the third day of training (*p* < 0.01, 95% CI: 12.5–70); rats exposed to 500 *μ*g/kg/day of MeHg learned the task by the fourth day of training (*p* < 0.05, 95% CI: 6.5–98.3); MK-801-treated animals did not learn the task (supplementary data). This finding suggests that MeHg exposure subtly disrupts learning acquisition or memory retrieval assessed at PDN 90. No differences were found for the retention test that was performed one week after the last training session (data not shown).  Table 3 shows the *F*-statistics and *p* values from two-way ANOVA analyses for MWM at PND-40 and PND-90.

The time-course analyses for the MeHg 250 *μ*g/kg/day groups indicated significant differences at test days 4 and 5 between the PND-40 and PND-90 groups. Thus, the detrimental effects on spatial learning detected in the group evaluated at PND-40 were not detectable in the group evaluated at PND-90. In the 500 *μ*g/kg/day MeHg-treated groups, the statistical analyses of escape latency at the two developmental windows studied showed that effects presented at test days 3 through 5 in the group evaluated at PND-40 were not presented in animals evaluated at PND-90 (Table 2).

### 3.4. Methylmercury Exposure Disturbed Acquisition and Extinction of Aversive Memories

**Figure 5:**
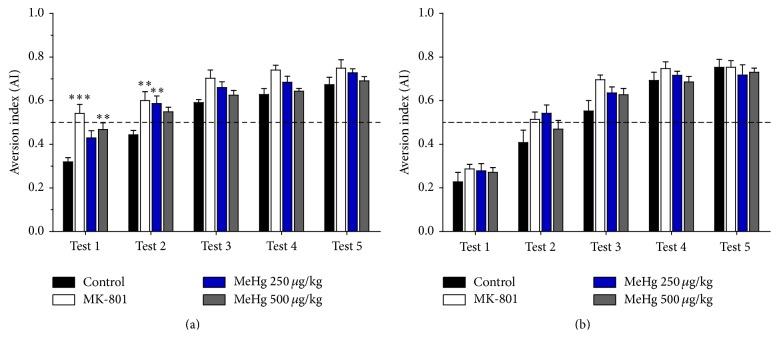
Methylmercury exposure disturbed the acquisition of aversive memories. The aversion index (AI) (mean ± SEM) of rats at PND-40 (a) and PND-90 (b). The data were analysed using two-way ANOVA followed by Bonferroni post hoc tests; significant differences compared with the control group are indicated (^*∗∗*^ *p* < 0.01; ^*∗∗∗*^ *p* < 0.001).

The CTA task evaluates the acquisition and extinction of aversive memories, as assessed by the AI. Water deprivation did not affect the body weights of the animals (data not shown). In animals assessed at PND-40, the control group had an AI of 0.3 on test day 1, which reflects aversion to saccharin (Figure 5(a)). The MK-801-treated animals had an AI of 0.54, indicating greater consumption of saccharin than the control group (*p* < 0.001, 95% CI: 0.07–0.3), representing no aversion to saccharin on Test 1. Animals exposed to 250 *μ*g/kg/day of MeHg demonstrated a slight increase in saccharin consumption (AI of 0.43), whereas animals exposed to 500 *μ*g/kg/day of MeHg did not decrease saccharin intake (AI of 0.47, *p* < 0.01, 95% CI: 0.02–0.27), reflecting no acquisition of aversion (Figure 5(a)).

In animals assessed at PND-90, no significant differences in AI were observed between groups on tests 1 through 5 (Figure 5(b)), indicating that, at this age, there was no effect of MeHg treatment on the acquisition of aversive memories.  Table 3 shows the *F*-statistics and *p* values from two-way ANOVA analyses for CTA at PND-40 and PND-90.

The time-course analyses for the 250 *μ*g/kg/day MeHg-treated groups indicated significant differences on Test 1, but none of those groups (PND-40 and PND-90) were different from their respective control. At PND-40, rats exposed to 250 *μ*g/kg/day of MeHg showed a slight, but not significant, preference for saccharin, suggesting no aversion. In contrast, animals assessed at PND-90 demonstrated an aversion to saccharin consumption (Table 2).

The time-course analyses for MeHg 500 *μ*g/kg/day indicated significant differences on Test 1 (*p* < 0.001) between the two assessed ages. Moreover, in this case the AI at PND-40 was significantly higher than that of the control group, which indicates no aversion to saccharin (Table 2) or a poor CTA acquisition. In contrast, the animals assessed at PND-90 demonstrated an aversion to saccharin, which indicates the acquisition of CTA.

## 4. Discussion

Despite knowledge of the adverse effects on the CNS caused by exposure to high doses of MeHg, there is a gap in knowledge of the detrimental effects of low and continual MeHg exposure during gestation and early postnatal life. The majority of studies evaluated the outcomes at one developmental stage, in some cases finding effects that were not assessed later in life, leaving as an open possibility the amelioration or worsening of the observed effects.

Epidemiological studies conducted in fish-eating populations have suggested that low and continual MeHg exposure could lead to behavioural outcomes [4, 5, 38]. Information on the possible adverse cognitive and behavioural effects of low-dose MeHg (500–1,000 *μ*g/kg/day) exposure is limited; the available findings are not conclusive, though these findings suggest a broad range of behavioural alterations including depressive-like behaviour [18].

Therefore, the study of exposure to environmentally relevant doses of MeHg is of special interest; the present study focused on the effects of this type of exposure from gestation through weaning on learning and memory and possible differential age effects. This study showed that early developmental exposure to doses of 500 *μ*g/kg/day of MeHg impaired exploratory behaviour, recognition memory, spatial learning, and aversive memories in periadolescent rats but not young adult rats. These findings could be explained by several factors such as the following: (a) the MeHg dose received by the pups was not sufficient to cause long-lasting adverse effects and compensatory systems could act to overcome the detrimental effects observed at PND-40; (b) the effects observed at PND-40 may be due to alterations in neurotransmission or synaptogenesis; (c) the younger CNS is more vulnerable to detrimental effects of MeHg.

Results from the OFT in periadolescent and young adult rats suggest that, at the doses and the exposure schedule used, MeHg did not induce detectable locomotor deficits in the offspring, which is consistent with the literature [18]. However, the locomotor activity of the group of rats that was exposed to 500 *μ*g/kg/day of MeHg was lower than the activity of the group that was exposed to 250 *μ*g/kg/day of MeHg, which suggests that a slightly higher dose than the dose used in this study might produce locomotor effects under our exposure protocol.

In the group that was exposed to 500 *μ*g/kg/day of MeHg, there was significant impairment in exploratory behaviour (rearings) on PND-40 but not on PND-90. The data from PND-40 are consistent with the literature [39, 40], and the age-dependent nature of the effect suggests a subtle alteration in the cerebellum, which is not strong enough to remain after the exposure is discontinued, when compensatory mechanisms could counteract the effect. MeHg has been reported to impair glutamatergic signalling in cerebellar granule cells [41], which could explain the alterations observed in exploratory behaviour.

In the animals assessed at PND-40, MeHg exposure had a dose-dependent effect on recognition memory. However, in the animals assessed at PND-90, no significant differences were detected for any dose of MeHg compared to the control group. In this regard, the potential detrimental effects of prenatal low-dose MeHg exposure are controversial. The epidemiological evaluation of the effects of prenatal MeHg exposure has been related to attention impairments in Faroese and Nunavik children [38, 42–44]. Moreover, in a U.S. cohort, visual recognition memory scores were improved in the infants of women who had higher levels of fish consumption, but the scores were lower when they were adjusted for hair mercury levels above 1,200 *μ*g/kg [45]. In contrast, visual recognition memory was not affected by prenatal MeHg in a Seychelles cohort of children evaluated at different ages [46, 47]. However, subsequent studies of the same cohort did not refute the later presence of delayed adverse effects in these children, possibly due to a restart of the exposure or the adjustment for relevant confounders [48]. These epidemiological and experimental findings confirm the complexity of prenatal MeHg exposure and the onset of cognitive effects.

Remarkably, the MWM data indicated that exposure to both doses of MeHg impaired spatial learning in animals trained at PND-40, but not in animals trained at PND-90. However, the normalized learning curve of the exposed animals was slower than that of control rats. These data suggest that exposure to doses as low as 250 *μ*g/kg/day of MeHg during CNS development impairs the acquisition of spatial learning, without abolishing the learning process. These findings suggest that early developmental exposure to low-doses of MeHg could be detrimental to the acquisition process, as demonstrated by the learning curve of the animals exposed to MeHg. The hippocampus plays a central role in correct performance on the MWM, and hippocampal vulnerability to MeHg has been reported at low-dose exposure (600 *μ*g/kg, PND-7), yielding reduced hippocampal neurogenesis and consequent memory disturbances in adolescent rats [49]. It is interesting that the MWM learning curve observed by Sokolowski et al. [49] did not suggest a learning deficit* per se* but did insinuate a learning disadvantage. This result is in agreement with our finding that, at young adulthood, the MeHg-exposed animals learned the task but at a slower rate than control animals. Experimental evidence for the effects of MeHg on spatial learning is contradictory [24, 40, 50], and it is difficult to compare across studies due to broad differences in the schedules of exposure and administered doses.

To our knowledge, this is the first study to assess the possible effect of MeHg on aversive memories. The CTA is assessed based on the conduct exhibited by the animal when it is challenged with a new taste; when the new flavour (conditioned stimulus) is associated with an ailment (unconditioned stimulus), the animal should develop an aversion to this flavour, and the animal should reject consumption of this flavour when it is presented again [51]. The brain structures involved in taste memory are the insular cortex [52, 53] and the amygdala [54]; it has been proposed that cholinergic signalling is mostly related to safe taste memories, whereas glutamatergic signalling is closely related to aversive memories [52–54]. We found that animals exposed to 500 *μ*g/kg/day of MeHg and trained at PND-40 showed no aversion to saccharin, suggesting a disruption in CTA acquisition, which could be due to impairment in glutamatergic signalling in the amygdala. In contrast, the MeHg-exposed animals that were trained at PND-90 demonstrated an aversion to saccharin, indicating no disturbances in CTA acquisition. However, the extinction rates for the MeHg and MK-801 groups were faster than for the control group, which suggests that although these treatments were not able to attenuate aversion, as occurred with animals trained at PND-40, the aversive memories were easily forgotten. Interestingly, the MeHg effects on CTA were dose- and age-dependent, which suggests that this task was useful to evaluate the behavioural effect of gestational exposure to low-doses of MeHg. Kakita et al. [30] reported that gestational exposure to 1,000 *μ*g/kg/day of MeHg decreased the neuronal population in the amygdala. In addition, amygdala functions are importantly related to anxiety behaviour, a characteristic that is related to low-dose prenatal MeHg exposure [18]. These effects should be studied further, particularly because no other study has assessed the effect of MeHg on aversive memories.

Overall, it is notable that the low-dose MeHg exposure was associated with adverse effects in the ORT, MWM, and CTA at PND-40, but at PND-90 only the spatial learning disturbance was observed. These findings could be due to an increased sensitivity or a preferential distribution of MeHg to the hippocampus. This brain region has been shown to be a potential target of MeHg accumulation [55] and its role in MWM performance has been widely studied. Thus, the results of this study point to the perirhinal cortex, hippocampus, and amygdala as brain areas that might be targets of gestational exposure to low-doses of MeHg because correct performance on the ORT, MWM, and CTA depends principally, although not exclusively, on these regions.

Our results for recognition memory, spatial learning, and aversive memories suggest a dose-dependent effect and consistently showed an age-dependent effect. That is, the MeHg effects are smaller when more time has passed between the last exposure and the evaluation of learning and memory. This could be due to the toxicokinetics of MeHg in the rat, resulting in reduced MeHg levels in the brain when exposure is terminated [26, 36]. Hu et al. [36] demonstrated that after gestational exposure ended, the levels of Hg in the brains of rats peaked 18 days after the last administration; a decrease in Hg levels began one week after that, and Hg was almost undetectable 40 days after the exposure ended. In our study, exposure stopped at weaning, but the postnatal exposure was received through maternal milk, which promoted limited transport of Hg [26], permitting the MeHg brain levels to decrease to undetectable levels at PND-90. However, in other studies, even when MeHg was no longer detectable in the brain, the effects of previous exposure were permanent [24], most likely due to damage caused by high doses of MeHg (5,000 *μ*g/kg MeHg) when it was present, such as cell death or inhibition of neuronal migration. Given the wide exposure schedule, there are different vulnerability windows of CNS development that could be disrupted by MeHg exposure [56]. The lowest level of exposure for which MeHg has been found to decrease DNA synthesis and neuronal number is in the range of 3,000 to 5,000 *μ*g/kg [24, 57]. However, at the doses used in this study, the observed effects would depend on the presence of MeHg, which has been reported to produce neurotransmission or synaptogenesis disturbances [56] associated with effects on learning and memory. Additionally, the differential age effects observed could be related to the increased susceptibility of younger organisms to deleterious effects of MeHg in the glutamatergic [58] and dopaminergic system [59], as well as to reactive oxygen species as reviewed in Farina et al. [60].

Although the experimental evidence indicates that the effects of developmental low-dose exposure to MeHg are subtle, it is important to mention that epidemiological studies have found detrimental effects on attention, learning, and memory in children exposed to “safe” levels of MeHg [61] at 7 years of age. These findings are in agreement with our results, suggesting that exposure to low-doses of MeHg is detrimental to cognitive processes at young ages. However, further studies are needed to determine whether the age-dependent “subtle” effects found in our study are also age-dependent in environmentally exposed populations.

It was notable that MK-801 (used to impair cognition) produced more pronounced effects at PND-40 than at PND-90. The differences in the animals' behaviour may be related to age differences or the treatment schedule as has been demonstrated by Nilsson et al. [62], who reported that MK-801 treatment does not increase the RI of animals.

## 5. Conclusions

Taken together, our results suggest that developmental low-dose exposure to MeHg disrupts memory and learning processes in a dose- and age-dependent manner and that if exposure stops at weaning, the effects observed at early life stages (PND-40 in rats, comparable to periadolescence in humans) will be subtle in later life (PND-90 in rats, comparable to young adulthood in humans). It is important to note that even when the exposed animals learned the different tasks in this study at PND-90, there was a delay in the correct performance of the MWM, which suggests a slight impairment in the acquisition of spatial learning.

In this study, the detrimental effects on behaviour were age-dependent, most likely because the exposure to low-doses of MeHg was stopped after weaning; however, in human populations exposed to MeHg, exposure does not stop at weaning, and, consequently, the adverse effects could be stronger than the effects observed in this study. The relevance of stopping exposure to MeHg after weaning in humans remains to be determined. These results may open a research field that could have relevance to establishing strategies to reduce the impact of* in utero* exposure to MeHg on learning and memory in humans.

## Supplementary Material

Supplementary material illustrates in figure 1 show the non-normalized effects of the methylmercury on spatial learning (escape latency) at PND-40 and PND-90 comparing different experimental conditions. The differences in escape latency were analysed using one or two-way ANOVA followed by Bonferroni post-hoc test.
Supplementary in figure 2, show the effects of the methylmercury on spatial learning differences within experimental group. Graphs show learning curve (mean ± SEM) by experimental group, normalized as a percentage of the latency of the group on the first day of training at PND-40 and PND-90. One-way ANOVA followed by Bonferroni post hoc tests were performed to analyse differences within experimental group.

## Figures and Tables

**Table 1:** The numbers and distributions of animals for each task and evaluation times.

	OFT	ORT	MWM	CTA	
Control	*n* = 10	*n* = 10	*n* = 10	*n* = 8	PND-40
	*n* = 10	*n* = 10	*n* = 10	*n* = 8	PND-90
MK-801	*n* = 10	*n* = 10	*n* = 10	*n* = 8	PND-40
	*n* = 10	*n* = 10	*n* = 10	*n* = 8	PND-90
MeHg 250 *μ*g/kg/day	*n* = 10	*n* = 10	*n* = 10	*n* = 8	PND-40
	*n* = 10	*n* = 10	*n* = 10	*n* = 8	PND-90
MeHg 500 *μ*g/kg/day	*n* = 10	*n* = 10	*n* = 10	*n* = 8	PND-40
	*n* = 10	*n* = 10	*n* = 10	*n* = 8	PND-90

**Table 2:** The effects of developmental exposure to MeHg on learning and memory in periadolescent and young adult rats.

	MeHg 250 *μ*g/kg/day			MeHg 500 *μ*g/kg/day		
	PND-40	PND-90	*p* value	PND-40	PND-90	*p* value
	(mean ± SD)	(mean ± SD)		(mean ± SD)	(mean ± SD)	
OFT^a^						
Distance (cm)	2512 ± 336.40	2536 ± 301.60	0.868	2118 ± 293.30	2371 ± 301.70	0.073
Rearings (times)	14.10 ± 2.42	13.70 ± 2.16	0.701	12.90 ± 1.97	13.10 ± 1.91	0.820
ORT^a^						
(recognition index)	0.557 ± 0.08	0.470 ± 0.14	0.154	0.3921 ± 0.126	0.5632 ± 0.110	**0.018**
MWM^b^						
(% escape latency)						
Day 1	100.0 ± 25.59	100.0 ± 19.93	ns	100.0 ± 30.84	100.0 ± 32.46	ns
Day 2	87.44 ± 47.29	79.69 ± 29.36	ns	77.85 ± 33.29	81.98 ± 43.48	ns
Day 3	74.04 ± 51.65	58.96 ± 44.11	ns	70.83 ± 47.24	57.61 ± 37.76	**<0.05 **
Day 4	80.43 ± 55.34	39.62 ± 54.37	**<0.01 **	64.82 ± 47.38	47.58 ± 35.85	**<0.01**
Day 5	67.10 ± 57.01	28.37 ± 51.32	**<0.01**	49.55 ± 42.78	30.23 ± 19.77	**<0.001**
CTA^b^						
(aversion index)						
Test 1	0.4295 ± 0.09	0.2781 ± 0.09	**<0.01 **	0.4681 ± 0.09	0.2714 ± 0.06	**<0.001**
Test 2	0.5869 ± 0.10	0.5418 ± 0.11	ns	0.5488 ± 0.06	0.4691 ± 0.11	ns
Test 3	0.6602 ± 0.07	0.6355 ± 0.08	ns	0.6254 ± 0.06	0.6274 ± 0.08	ns
Test 4	0.6844 ± 0.08	0.7161 ± 0.05	ns	0.6435 ± 0.04	0.6859 ± 0.07	ns
Test 5	0.7282 ± 0.05	0.7173 ± 0.13	ns	0.6907 ± 0.06	0.7302 ± 0.05	ns

OFT: open field test; ORT: object recognition test; MWM: Morris water maze; CTA: conditioned taste aversion.

^a^Results from one-way ANOVA; ^b^Results from two-way ANOVA.

**Table 3:** *F*-statistics and *p* values from statistical analyses for the OFT, ORT, MWM, and CTA in periadolescent (PND-40) and young adult rats (PND-90).

Task	PND-40		PND-90	
	*F*-statistic	*p* value	*F*-statistic	*p* value
OFT^a^, distance	*F*_3,36_ = 5.7	0.0027	*F*_3,36_ = 9.6	<0.0001
OFT^a^, rearings	*F*_3,36_ = 6.8	0.0009	*F*_3,36_ = 10.6	<0.0001
ORT^a^	*F*_3,36_ = 9.9	0.0002	*F*_3,36_ = 4.5	0.0095
MWM^a^				
Control	*F*_4,45_ = 11.32	<0.0001	*F*_4,45_ = 17.31	<0.0001
MK-801	*F*_4,45_ = 0.7188	0.5841	*F*_4,45_ = 1.997	0.1134
MeHg 250 *µ*g/kg/day	*F*_4,45_ = 0.6753	0.6126	*F*_4,45_ = 18.71	<0.0001
MeHg 500 *µ*g/kg/day	*F*_4,45_ = 1.641	0.1858	*F*_4,45_ = 6.344	0.0004
MWM^b^,				
Interaction	*F*_12,1880_ = 6.521	<0.0001	*F*_12,1880_ = 11.47	<0.0001
Time	*F*_4,1880_ = 73.20	<0.0001	*F*_4,1880_ = 252.8	<0.0001
Treatments	*F*_4,1880_ = 73.63	<0.0001	*F*_4,1880_ = 26.11	<0.0001
CTA^b^, aversion index				
Interaction	*F*_12,112_ = 1.004	0.4507	*F*_12,112_ = 1.95	0.0357
Time	*F*_4,112_ = 53.70	<0.0001	*F*_4,112_ = 307	<0.0001
Treatments	*F*_3,112_ = 13.60	<0.0001	*F*_3,112_ = 1.21	0.3258

OFT: open field test; ORT: object recognition test; MWM: Morris water maze; CTA: conditioned taste aversion.

^a^Results from one-way ANOVA; ^b^Results from two-way ANOVA.

## Abbreviations

CNS: Central nervous system
CTA: Conditioned taste aversion
GD: Gestational day
IQ: Intelligence Quotient
MeHg: Methylmercury
MWM: Morris water maze
NMDAR: N-Methyl D-aspartate receptor
OFT: Open field test
ORT: Object recognition test
PND: Postnatal day.
