# Supplementary material for: Early Developmental Low-Dose Methylmercury Exposure Alters Learning and Memory in Periadolescent but Not Young Adult Rats
Source: Biomed Res Int. 2016 Jan 13;2016:6532108. doi: 10.1155/2016/6532108 (PMC4738696; doi:10.1155/2016/6532108)
Supplement: Supplementary file 1 — Supplementary material illustrates in figure 1 show the non-normalized effects of the methylmercury on spatial learning (escape latency) at PND-40 and PND-90 comparing different experimental conditions. The differences in escape latency were analysed using one or two-way ANOVA followed by Bonferroni post-hoc test. Supplementary in figure 2, show the effects of the methylmercury on spatial learning differences within experimental group. Graphs show learning curve (mean ± SEM) by experimental group, normalized as a percentage of the latency of the group on the first day of training at PND-40 and PND-90. One-way ANOVA followed by Bonferroni post hoc tests were performed to analyse differences within experimental group. [file 6532108.f1.pdf]

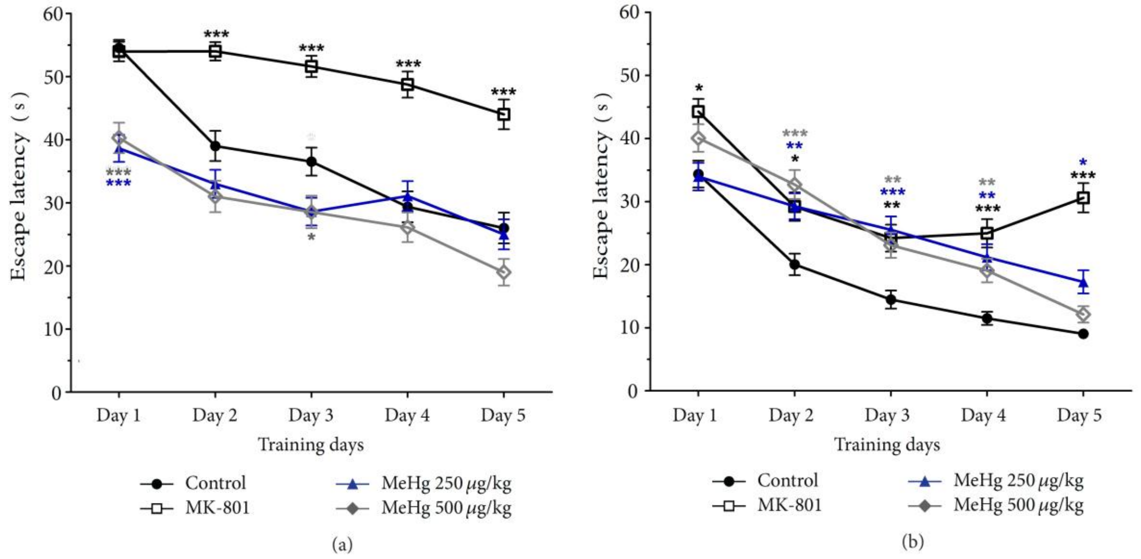

Supplementary figure 1. Methylmercury effect on spatial learning. Graphs show the learning curve of the five training days (mean ± SEM) as escape latency (s) performed at PND-40 (a) and PND-90 (b). Escape latency results were analysed using two-way ANOVA, followed by Bonferroni post hoc tests; statistically significant differences compared with the control group are indicated (*p<0.05; **p<0.01; ***p<0.001).

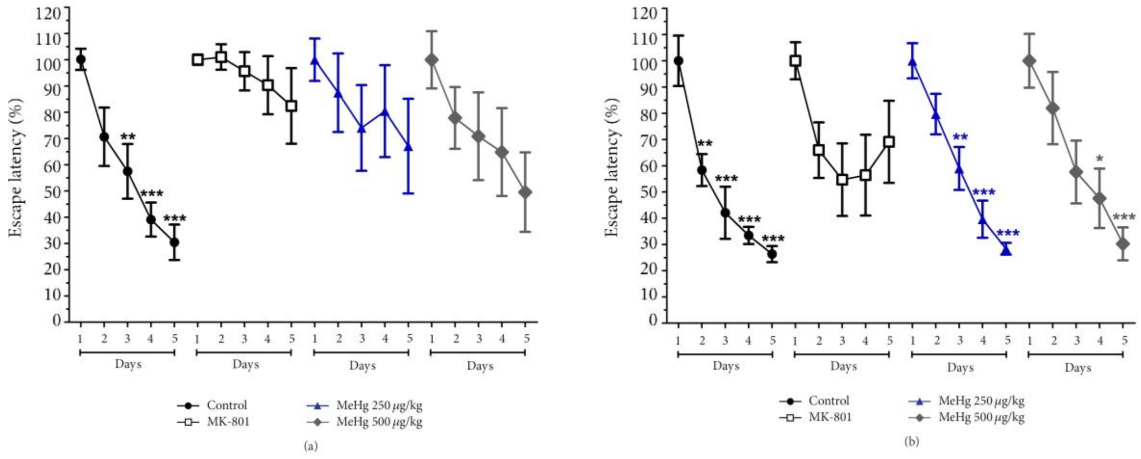

Supplementary figure 2. Spatial learning differences within experimental group. Graphs show learning curve (mean ± SEM) by experimental group, normalized as a percentage of the latency of the group on the first day of training at PND-40 (a) and PND-90 (b). One-way ANOVA followed by Bonferroni post hoc tests were performed to analyse differences within experimental group. Statistically significant differences are indicated (*p<0.05; **p<0.01; ***p<0.001).
